# Personalization and localization as key expectations of digital health intervention in women pre- to post-pregnancy

**DOI:** 10.1038/s41746-023-00924-6

**Published:** 2023-09-30

**Authors:** V Vien Lee, Smrithi Vijayakumar, Wei Ying Ng, Ni Yin Lau, Qiao Ying Leong, Delicia Shu Qin Ooi, Lin Lin Su, Yung Seng Lee, Shiao-Yng Chan, Agata Blasiak, Dean Ho

**Affiliations:** 1 The Institute for Digital Medicine (WisDM), Yong Loo Lin School of Medicine, National University of Singapore, Singapore, Singapore; 2 The N.1 Institute for Health, National University of Singapore, Singapore, Singapore; 3 Department of Paediatrics, Yong Loo Lin School of Medicine, National University of Singapore, Singapore, Singapore; 4 Division of Paediatric Endocrinology, Department of Paediatrics, Khoo Teck Puat-National University Children’s Medical Institute, National University Hospital, National University Health System, Singapore, Singapore; 5 Department of Obstetrics & Gynaecology, Yong Loo Lin School of Medicine, National University of Singapore, Singapore, Singapore; 6 Department of Obstetrics & Gynaecology, National University Hospital, Singapore, Singapore; 7 Singapore Institute for Clinical Sciences, Agency for Science, Technology and Research (A*STAR), Singapore, Singapore; 8 Department of Biomedical Engineering, College of Design and Engineering, National University of Singapore, Singapore, Singapore; 9 Department of Pharmacology, Yong Loo Lin School of Medicine, National University of Singapore, Singapore, Singapore; 10 The Bia-Echo Asia Centre for Reproductive Longevity and Equality, Yong Loo Lin School of Medicine, National University of Singapore, Singapore, Singapore

**Keywords:** Patient education, Health care

## Abstract

Health behaviors before, during and after pregnancy can have lasting effects on maternal and infant health outcomes. Although digital health interventions (DHIs) have potential as a pertinent avenue to deliver mechanisms for a healthy behavior change, its success is reliant on addressing the user needs. Accordingly, the current study aimed to understand DHI needs and expectations of women before, during and after pregnancy to inform and optimize future DHI developments. Forty-four women (13 pre-, 16 during and 15 postpregnancy; age range = 21–40 years) completed a 60-minute, semistructured, qualitative interview exploring participant’s experience in their current phase, experience with digital health tools, and their needs and expectations of DHIs. Interviews were audio-recorded, transcribed verbatim and thematically analyzed. From the interviews, two core concepts emerged—personalization and localization of DHI. Between both concepts, five themes and nine subthemes were identified. Themes and subthemes within personalization cover ideas of two-way interactivity, journey organization based on phases and circumstances, and privacy trade-off. Themes and subthemes within localization cover ideas of access to local health-related resources and information, and connecting to local communities through anecdotal stories. Here we report, through understanding user needs and expectations, the key elements for the development and optimization of a successful DHI for women before, during and after pregnancy. To potentially empower downstream DHI implementation and adoption, these insights can serve as a foundation in the initial innovation process for DHI developers and be further built upon through a continued co-design process.

## Introduction

Digital health interventions (DHIs) are an emerging stream of digital technologies used to deliver a range of health services and treatments, including interventions for patients, healthcare providers, health system and data services^1^. Given their potential for scalability and accessibility, DHIs have been increasingly used for health promotion and improving health outcomes for chronic conditions through multi-faceted strategies, including education, peer connection, titration of medication, health status monitoring, remote access to therapies and patient-provider communication^2^. Despite the heterogeneity of the DHI modality, systematic reviews have generally reported promising clinical benefits in the management of various chronic conditions, including cardiovascular disease, musculoskeletal pain, functional disability, and mental health conditions^3–5^. Among nonclinical populations, DHIs have been shown to facilitate healthy behavior change in a variety of domains, including sleep, physical activity, diet and tobacco use^6–8^.

Pregnancy is a period of transition that involves rapid physiological, emotional and behavioral changes. Even in uncomplicated pregnancies, these changes can have lasting effects on maternal and infant health outcomes^9^. Based on findings from the Growing Up in Singapore Towards healthy Outcomes (GUSTO) birth cohort study, a range of maternal characteristics and behaviors (e.g., dietary pattern, nutrient intake, weight status, mental health status) during and after pregnancy can impact physical, neurobehavioral and socioemotional development of the child^10–13^. Using the heuristic model of McBride et al.^14^ for a teachable moment, Phelan called attention to pregnancy as an ideal stage to motivate individuals to adopt risk-reducing health behaviors due to the intersection of significant cueing events during this time period (i.e., increased perceived risk outcomes of own and baby’s health, strong emotional response towards the well-being of the baby, and redefinition of self into a maternal role)^15^. Accordingly, this presents a timely opportunity to intervene and cultivate healthy behavior changes.

Given the impact of lifestyle choices on maternal and child health outcomes, and the suitability of DHI to drive behavioral change, the majority of current DHIs for women during and postpregnancy focus on strategies for lifestyle modification, including diet, physical activity, and weight management^16,17^. Nevertheless, a recent review of DHI for diet and physical activity during pregnancy reported mixed findings, with only three out of seven studies reporting significant effects of the intervention on physical activity, and only one study out of six reporting an improvement in diet^16^. Notably, studies that observed no effect of DHI also reported poorer levels of engagement (i.e., consistent usage of digital health tool throughout the intervention period), with one study reporting frequent DHI usage patterns being associated with better intervention outcomes (i.e., lower gestational weight gain)^16,18^.

An increasingly used method to boost end-user digital health engagement is to adopt the principle of co-design when developing digital health technologies. Co-design involves a shift of process from expert-led or technology-driven development work where interventions are designed *for* end-users to one where they are designed collaboratively *with* end-users^19^. Co-design advocates the involvement of users throughout the development life cycle of the DHI, with early stages focusing on an initial ‘discovery’ phase to identify user needs and preferences, followed by multiple iterative design cycles before narrowing down the focus onto prototype development^19^. Involving end-users in the initial innovation process can ensure that DHIs are relevant and acceptable while meeting the needs of the users, women in this case^20^. This approach is particularly relevant in the field of gynecology and obstetrics, where female perspectives have been traditionally underrepresented^21^. As such, the co-design process can become not only a facilitator of more effective technological solutions but also a step towards long-called for equity in women health sciences^22^.

The current study aims to interview women from three different phases—preconception, pregnancy, postbirth—to understand their DHI needs and expectations, and, in turn, inform future, equitable DHI development for healthy behavior change in women across these phases.

## Results

### Participant characteristics

A total of 44 participants (age range = 21–40; mean age = 31.6 years, SD = 4.0) completed the study. One hundred and twenty-five women indicated interest in the study but 81 were not enrolled in the study due to purposive sampling strategies (30 individuals), lack of response from participants after receiving the information sheet (22 individuals), and the study’s predetermined inclusion and exclusion criteria (3 individuals). Similar to Singapore’s ethnic distribution^23^, participants were of Chinese (75.0%), Malay (9.1%), Indian (9.1%) and other (6.8%) ethnicities, and all participants spoke fluent English. Participant demographic data based on their groups (preconception, pregnancy and postbirth) are presented in Table 1.

**Table 1:** Demographic data of study participants.

Demographics	Preconception	Pregnancy	Postbirth
N	13	16	15
Age range (years)	21–39	27–39	25–40
Socioeconomic status (n)			
Low	1	4	4
Middle	5	4	4
High	7	8	7
Ethnicity (n)			
Chinese	11	11	11
Indian	0	1	3
Malay	2	1	1
Others	0	3	0
Number of children (n)			
0	7	10	0
1	5	4	7
2 or more	1	2	8
Education (n)			
15 years or less	7	10	5
More than 15 years	6	6	10

Current technology usage of participants is shown in Table 2. Two participants (one from pregnancy and one from postbirth) were excluded from the table due to missing data from uncompleted post-interview questionnaires. Participants across the three phases used a variety of health-related mobile phone apps, including apps for physical health, mental health, fertility, pregnancy and babies. The study population reported using a median of 2 (interquartile range = 2.0) apps, with a range of 0–9 different mobile phone apps. The internet was ranked as the first source of information by majority of the participants and all participants reported using the internet to find fertility, pregnancy or child-related information.

**Table 2:** Technology usage of study participants (*N* = 42).

Technology usage	Preconception	Pregnancy	Postbirth
Ranked first for source of information [out of 7; *n* (%)]			
Internet	8 (61.5)	7 (46.7)	9 (64.3)
Mobile phone apps	1 (7.7)	0 (0.0)	1 (7.1)
Social media	1 (7.7)	1 (6.7)	1 (7.1)
Healthcare providers	1 (7.7)	7 (46.7)	1 (7.1)
Alternative care providers	0 (0.0)	0 (0.0)	0 (0.0)
Family	1 (7.7)	0 (0.0)	1 (7.1)
Friends	1 (7.7)	0 (0.0)	1 (7.1)
Number of apps used			
Median (Interquartile range)	2 (2.0)	2 (2.0)	1 (2.5)
Range	0–5	0–4	1–9
Mobile phone application usage			
Physical well-being health apps	Apple Health, Fitbit, Samsung Health, Poop diary	AIA Vitality*, Fitbit, NBuddy*	Calm: Sleep, Google Fit, Meditation, MyFitnessPal, Prenatal Yoga Down Dog, Samsung Health, Upside Motion
Government health apps	HealthBuddy*, HealthHub*, Healthy365*, Lumihealth*	Healthy365*	HealthBuddy*, HealthHub*, Healthy365*
Mental health apps	Headspace, Mindfulness apps	–	–
Fertility or pregnancy or baby-related apps	Baby Read, Fertility Friend, MyFertiliy,	BabyCenter, Mama & Baby, Pregnancy + , Pregnancy After Loss, The Asian Parent*, What to Expect, Wonder Weeks	BabyCenter, BabySparks, Baby Tracker, Huckleberry, Little Family Room*, Pregnancy, The Asian Parent*, The Wonder Weeks, What to Expect
Fertility or pregnancy or baby-related trackers	Flo, Menstruation calendar, Ovia, Ovia Pregnancy, Period trackers	Babytracker, Count the Kicks, Flo, Ovia	BabyTracker, Count the Kicks, FeedBaby, Flo, Ovia, Ovia Pregnancy and Baby Tracker, Period Tracker, Pregnancy Tracker, Pump Log

*locally-developed apps.

### Interview data

From the interviews, two core concepts emerged—personalized journey and localization of DHI. Personalized journey describes preferences for digital health personalization, while localization of DHI describes preferences for local relevance in digital health tools. Table 3 provides additional quotes from all three groups for each theme and subtheme.

**Table 3:** Additional quotes from all three groups (preconception, pregnancy and postbirth) for each theme and subtheme.

Themes & Subthemes	Preconception	Pregnancy	Postbirth
**Personalized Journey**			
*Two-way interactivity*			
Data-driving personalized interaction	“If I can key in the number of hours I sleep, the type of exercise I do, what I eat… they will generate like a checklist of things that you need to eat… to boost up the health.” -Participant 5	“It would be nice to track for example, my weight loss - this is how much I did weigh, and these are the results [for] glucose levels, glucose tests, urine tests… because I lost 18 pounds in the first month, I am wondering should I be concerned.” -Participant 6	“The personalization must grow as our baby grows. So [with each] milestones achieved, maybe suggestions of activities to do at home, places to bring them to.” -Participant 11
Right timing	“Just show me whatever information is relevant to me now. Don’t show me what to do when you’re pregnant… If I were to use an app, I’d rather just use the stuff that’s relevant to me.” -Participant 35	“They tell you to go for activities which are less strenuous. During the first trimester, try not to exercise too much. To a certain extent, there should be a bit more information about what exercises can be done during the pregnancy itself.” -Participant 33	“There’s chat for the [app], so it’ll send out notifications from other mummies. It starts to give out notifications [that is] unrelated [so,] you don’t want to see at all. I find it quite annoying.” -Participant 17
*Journey organization*			
Milestone indication	“Maybe you can… tell people like okay, now you are at like 31 years old, you should be doing this [fertility] check if you have not done it within the past 2 years or 3 years. So, it serves as a reminder for them.” -Participant 39	“It would be useful to show how the pregnancy is at every week… like what you can see at 28 weeks of pregnancy… how the pregnancy is progressing.” -Participant 29	“Their milestones like gross motor milestones, fine mental milestones are not consolidated in one app… don’t think I have seen it in a specific place. Maybe that would be helpful for parents who are not aware.” -Participant 26
*Privacy trade-off*			
Extent of information collection	“I would like [feedback]. What I think I need, may not be what I actually need. But if you are talking about algorithm-based then it’s going to be very difficult, and there is a lot of data sharing involved. -Participant 41	“[Personalization will be great, but I don’t know whether it is going to be possible and how much of a personal information I need to give in order for it to be personalized on me for me.” -Participant 31	“Personally, because I’m [have a] scientific background, I know that these factors (referring to age and due date) play a very important role in the health of the mother and the baby… So, I don’t mind giving [that] information.” -Participant 44
Trust in government and hospitals	“Must see which organization, if it’s the government, then it’s okay. If it’s a private one, then no, [I] won’t.” -Participant 36	“At the end of the day, they can give you the information and I will decide… at least it’s like having someone to [find] the information. Okay, this is what [the app] found and I will literally follow the healthcare.” -Participant 31	“It depends for what purposes, how, and who the data goes to, how it’s being stored. Let’s say it’s for Singpass, I would give because it’s a governmental database and it’s a reliant source.” -Participant 8
**Localization of DHI**			
*Local health-related resources*			
Access to local healthcare resources	“Notifications… with a localized context. Like say, are you like a homemaker…. maybe you can have like express therapy [for] half an hour, next door.” -Participant 12	“People in your community or area that’s specialize in pre- [or] post-natal would be good. Okay, you’re in the west of Singapore, here’s a great therapist or counsellor [or] physio.” -Participant 7	“It was a Saturday [and] I couldn’t find information on which clinic I should bring him to for an x-ray. It took me so long to find the information because the polyclinic was not available… on weekend[s].” -Participant 24
Understanding the local healthcare process	“Let’s say if I’m pregnant, I have no idea what to do. Should I book an appointment with a doctor? NUH (local public hospital) or private? Which direction should I go to? Is it find a doctor first? Or choose a hospital first? So these kind of things I’m not too sure.” -Participant 30	“When should I like book my maternity tour with [the] private hospital that I want? When should I even check into the hospital? So, things like appointment scheduler would be helpful and for Singapore.” -Participant 4	“There’s really quite a lot of stuff that you have to do after you have a baby, such as churning out the birth cert, applying for your baby bonus, all that. I wonder if there could be a one-stop app to kind of guide you through all the things that you have to do because being a parent can be quite overwhelming and you might not have the headspace to think about all these things.” -Participant 23
Localized lifestyle guidance	“I don’t use [apps], but I will go through to see what type of Traditional Chinese Medicine they recommend, which gynae they go to [and] what type of journey that they had.” -Participant 36	“Sometimes I [wonder], can I eat black sesame? Online they only tell you about sesame seeds [but] they don’t tell you about black sesame paste… so you don’t know whether is okay.” -Participant 15	“Having a more localized version that’s developed by a team that is either based here or knows the food here will help… like I try to type char kway teow (local dish), I can find it. Versus like say a Google Health app, that will be less likely… it’s very hard to find certain food that we eat here.” -Participant 18
*Hearing from a local voice*			
Connecting with a community through personal anecdotes	“I’m all for [connecting with others], if only people would be willing to talk about it… I think, this kind of connection can help heal [because] you [don’t] need to struggle alone, that’s what your app is supposed to do.” -Participant 32	“I’d like to just be able to talk to moms about like getting the vaccine… I was able to text the group [from school and] that piece of reassurance of 3 moms responded right away and said - Oh my gosh, I’m thinking the same thing just today.” -Participant 6	“When the mummies chat on [an] issue, they [will] ask - what do you do? I [will] tell my point of view and other mummies will suggest so, we pick up from there… So, when I told the mummy’s group [about my daughter’s cough], they said why not you try to go over the counter and purchase [cough medication] … So, I bought one and keep it at home.” -Participant 42

### Personalized Journey

Under the concept of personalized journey, three themes and five subthemes emerged from participants’ responses. The themes include two-way interactivity, journey organization and privacy trade-off.

### Two-way interactivity

#### Data-driven personalized interactions

When discussing digital health tools, participants consistently brought up tracking as a feature of interest. Prior to conception, the want for tracking revolves around women’s reproductive health to help with conception (e.g., period and ovulation tracking). During pregnancy, the priority shifts to pregnancy symptoms and weight gain or loss. Following birth, the focus of tracking further evolves from women-focused to child-focused (e.g., child sleep, feeding time, diaper change). Nevertheless, participants expressed that tracking can be stressful due to the need for strict compliance of data input and/or lack of time. Participant 14, who is trying to conceive, shared that “Having to comply to the app would be more stressful to me… doing [a stress diary] would cost more stress to me. Do a food diary? Nope, that’s going to be more stressful.”

When providing data to digital health tools for tracking (i.e., manual input or wearables), it is important to note that women expect feedback that is adaptive based on their input. Participants highlighted the need for feedback to help them better understand and troubleshoot their conception to postbirth journey. When asked about her tracking experience, Participant 14 shared, “If there is a function that would tell me… Okay, great job mommy! You only had [this] many calories… I don’t think there are any apps that help track… you are at this trimester and you gained this amount of weight, is this healthy or not? If it’s not healthy, here is a bunch of stuff you can do about it.” This was further expanded by Participant 3, who had irregular periods when trying to conceive, “If you have irregular menses, [what should] you do? So, I think [tracking] will be more useful when there is information for troubleshoot[ing].” Moreover, feedback can generate positivity in their journey and allow women to be more in tune with their bodies. For instance, Participant 39 expressed joy when her tracker accurately predicted her experiences while trying to conceive, “When I was using the tracker, I was quite happy. When the tracker said, okay now… this period you will experience… [this] type of discharge [and] if you spot it, [it’s] very accurate. So, these are the happy journey… you feel very connected when your body experiences match what the app says.”

Alongside adaptive feedback, women also value the ability to customize the interface according to their current priorities. For instance, Participant 8, a first-time mum, mentioned, “You can do like… a preference where you tick what would you like to see… would you like to see things on like more on baby-led weaning, or sleep training? Or more mental health [or] physical health? You can tick your preference and… have tips [come] up… according to your preference.”

#### Right timing

With the overwhelming amount of information participants received online or from friends and family, participants discussed the need to couple adaptive feedback with timeliness. Part of personalizing women’s digital health experience throughout the three phases involves providing timely feedback that is relevant to them at that point in time. This was explained by Participant 16, “One classic and very easy way is to do the day-by-day notification to say that your kid is currently at this stage… Technically, it is quite one-sided because you are just giving us information but you are giving me information that actually matters to me at that point. So, it’s very timely. At that point, it feels very personalized. So, therefore, people generally will feel it is more two-way instead of just one-way.”

As part of providing feedback at the right time, the majority of participants indicated a preference for receiving customized information based on the current phase they’re in (i.e., preconception, pregnancy or postbirth). While women prefer an all-in-one app that supports them throughout their journey from preconception to postbirth, information presented should be organized based on current needs. Beyond the longitudinal segmentation of phases, participants also highlighted the need for customization of support based on their current pregnancy or motherhood circumstances (e.g., first-time mums, pregnant with twins, premature birth). When talking about customized digital health support, Participant 7 suggested, “I guess kind of like pick a path, do you have a girl? Do you have a boy? Do you have twins? Are you in this age bracket? Was this the kind of birth you had? Knowing… these details [can] change [the information provided].”

### Journey organization

#### Milestone indication

As part of providing personalized support, participants highlighted the importance of providing milestone indicators relevant to their current phase. While trying to conceive, women would like to know suitable timelines for fertility checks and further medical investigations if required. Nevertheless, excessive reminders during this stage can be stressful as women prefer not to be constantly reminded that they are trying to conceive. Pregnancy and postbirth, milestone indicators should shift focus to baby’s development (e.g., fetal brain development, fine motor skills development). While most women understand that development guidelines provided may not be completely accurate to their situation, understanding their child’s milestone can be empowering through providing women with information for better behavioral decision making. This was summed up by Participant 16, “You take [the guidelines] with a pinch of salt, but you enjoy reading… knowing that this next 2 weeks, your baby’s brain is undergoing growth and so on… Whether you know [or] don’t know, [the information] clearly has no implication on your baby[’s growth] but [it] does help improve empowerment of the mom or the parents… I think it does contribute to [better] mental health for the mom… knowing is always better than not knowing.”

### Privacy trade-off

#### Extent of information collection

While the majority of participants expressed interest in a personalized digital health tool, participants were also aware of the amount information required to provide a deeper level of personalization (e.g., customized diet recommendations, troubleshooting of specific health issues) beyond surface-level customization. Participant 34 discussed, “[It depends on the] degree of what people are comfortable with, but if you want [the digital health tool] to be personalized then you have to give up your information… if not, you can’t expect such services.” While some participants were only comfortable to provide the basic, demographic information (e.g., age, ethnicity, estimated date of delivery), other participants perceived that a useful digital health tool would incorporate their medical health records into feedbacks and recommendations. For instance, Participant 16, who has gestational diabetes, proposed, “Definitely, I think it is necessary that patients’ medical records have to be incorporated into the algorithm. That will raise the issue of access… to the patients’ private information, but I don’t think that it’s even worth considering if you can’t personalize it based on that… It’s not going to be really useful, to be honest. For example, take myself as a case study, if the app cannot even retrieve the information on my blood glucose readings, how can it be that helpful?”

#### Trust in government and hospitals

Despite the reservations some participants had in regards to the extent of information collected, most of the participants were willing to provide more personal data if the digital health tool was developed or authorized by the government or their healthcare provider. This was further elaborated by Participant 43, “If you are my doctor and it’s coming from you, I’ll [have] more trust than… other platforms. If I’m going to NUH for 8 to 9 months, I have a different kind of trust and a different kind of assurance from NUH. There are lots of things that you can find [online] but if it’s coming from… my hospital, [I] will be more assured… it will be more reliable for the patients, rather than [having to go through] so many platforms and trusting anybody else.”

### Localization of DHI

Under the concept of localizing DHI, two themes and four subthemes emerged from participants’ responses. The themes include access to local healthcare resources and hearing from a local voice.

### Local health-related resources

#### Access to local healthcare resources

When asked about localized needs, participants indicated the importance of awareness of and access to local healthcare resources. Healthcare resources that women were interested in within their region include: local medical seminars, fertility testing, gynecologists, maternity ward options, pre- or post-natal physiotherapists, counsellors, lactation consultants, post-natal massage therapists and pediatricians. For instance, when asked about helpful features in digital health tools, Participant 7 described, “People in your community or area that specialize in pre- [or] post-natal would be good. [If] you’re in the west of Singapore, here’s a great therapist or counsellor [and] here’s a great person to do physio[therapist].” Apart from providing information on how to access local healthcare resources, participants expressed that they would like a centralized digital health tool from their hospital to connect them directly to all of the services that they’re engaged with, such as appointment scheduling, clinic registration, access to medical records and bill payment. Aligning digital health tools to a practical aspect of patients’ healthcare journey can facilitate uptake and continuous engagement. As described by Participant 22, “[For the digital health tool] to be aligned with… a practical aspect, like say a medical appointment because probably you wouldn’t miss [the appointment]. Then [the tool] could be tied to the medical appointment so… it makes usage more relevant.”

#### Understanding the local healthcare process

Besides access to local healthcare resources, participants discussed their struggles in understanding next steps when navigating through their healthcare journey from preconception to postbirth. Challenges often involved uncertainty about health check-ups, differences between public and private healthcare services, and administrative procedures. For instance, Participant 30, who is trying to conceive, shared, “Let’s say if I’m pregnant, I have no idea what to do. Should I book an appointment with a doctor? … public or private, which direction should I go to? Is it find a doctor first? Or is it choose a hospital first? So, these kinds of things I’m not too sure.” By using a digital health tool, participants would like to be equipped with a greater awareness and clearer expectations of immediate next steps (e.g., When should I do my fertility test? What to expect during my 32-week scan? What should I ask the pediatrician during my child’s first appointment?).

#### Localized lifestyle guidance

Participants also discussed the need to receive localized guidance relating to lifestyle, specifically diet and alternative medicine options. This factor can vary substantially across contexts, and as such, may require specific attention by DHI developers in order to achieve successful adherence and implementation outcomes. Participants often sought guidance regarding their diet options during pregnancy and confinement online, however, information on websites often did not include local options. Participant 41 pointed out, “If we are supposed to avoid fatty food or oily food, information from Western websites [will] probably [suggest] eat more grains. But for us, rice is the staple [so], what do you mean by eat more grains… If [the website] say eat a balanced diet, does that mean nasi lemak (*local rice dish known to have high calories and fat content*) is a balanced diet? If I eat nasi lemak every day, am I healthier?” When faced with traditional diet advice from family and friends, participants tended to comply despite being uncertain about its effectiveness. Equipping women with knowledge to navigate through unfounded advices can contribute towards empowerment in decision making about their lifestyle choices. Participant 16 shared, “I have this friend who, the mom was asking her to drink a lot of vinegar to help with the [nausea] but it didn’t work for her because it makes her want to throw up even more. But because the mom said so, she continued doing it, forcing herself and throw up again. So, [it] requires… re-education because you can be the one to provide information but it also requires [women to make up] their own mind.”

### Hearing from a local voice

#### Connecting with a community through personal anecdotes

When navigating through their journeys from preconception to postbirth, participants expressed the need to connect with other local women online (e.g., online forums, messaging apps, Facebook groups) from similar phases. By connecting with women in similar situations, participants were able to seek support for their struggles that partners or family might not understand, and reassurance regarding their concerns and worries. This need was conveyed by Participant 44, “[It] would really help if there’s some sort of initiative, [where] recent mothers [can] share their experience so that people who are pregnant and are going to deliver soon can read them and understand okay, this is what everyone’s going through. So, there’s no need to panic or anything… [pregnancy is] naturally [a] very stressful journey so, some sort of verbal support and virtual support matter a lot, even if they [are not] in-person.” Furthermore, the sense of reassurance can help normalize situations when a woman perceives her experience to be atypical (e.g., fertility issues, difficult pregnancy symptoms, difficulty with latching during breastfeeding). When asked about the usefulness of online support for polycystic ovary syndrome (PCOS), Participant 37 replied, “It normalizes the experiences. When you see that there are so many other people who have this issue because it is actually quite common, like one in ten, you feel like it’s normal. It does help.” Nevertheless, some participants highlighted their preference to stay anonymous when interacting with other women on digital health tools and will only share more personal information with a closer, smaller group.

Besides connecting with other women, participants expressed interest in receiving information through personal anecdotes, specifically from a local perspective. Participant 4 mentioned, “One thing that is helpful is birth journeys or pregnancy stories from other women in Singapore because US-based pregnancy might be different [from] us. Like how many [clinic visits] depends on the gynae or the entire experience might be different. So, having that Singapore-based perspective might be helpful.” Personal anecdotes that participants sought ranged from success stories to misfortunes and setbacks. While participants trusted healthcare providers to provide adequate medical care, participants conveyed that the use of similar language (e.g., lay terminology) by their peers is helpful and more approachable.

## Discussion

Our study identified user needs and expectations of DHI in women trying to conceive, currently pregnant and up to two years postbirth. Overall, participants in the current study had considerable experience interacting with health-related digital tools. They highlighted several needs that were not fulfilled by currently used digital health tools, and indicated the need for DHIs to be personalized and localized. DHI personalization should include two-way interactivity and customized journey while allowing individuals to control privacy trade-offs. DHI localization should include access to local healthcare resources, and local communities and women’s stories.

In line with existing studies exploring women’s expectations of pregnancy apps, personalization of digital tools has emerged as an important aspect for women when considering DHIs to support their maternal journey^24^. Besides improving adherence, personalized DHIs with tailored feedback have shown promising outcomes in promoting healthy behaviors (e.g., maintaining a balanced diet, preventing alcohol consumption) during pregnancy^16,25,26^. Nevertheless, when personalizing DHIs, participants cited surface-level personalization (e.g., inclusion of user’s name on the interface) to be an insufficient incentive to encourage continued DHI usage. While participants were interested in tracking their health behaviors, they were only inclined to sustain their tracking behavior if adaptive and timely feedback was provided. This is in line with an emerging intervention design, the just-in-time adaptive intervention (JITAI)^27^. JITAI aims to provide the right type and amount of support or actionable feedback, triggered by the system, at the precise timing needed to induce the desired change^27^. While research into JITAIs for health-related behaviors are in its early stages, a meta-analysis looking at JITAIs for a range of behavior of interests (e.g., healthy diet, mental health, addiction, weight loss) reported up to 0.87 times improvement on health outcomes of JITAI groups over alternative interventions^28^. Nevertheless, a recent systematic review reported that many JITAIs for physical activity lack evaluation of JITAI usage and adoption among representative real-world and clinical populations^29^. Accordingly, DHI innovators should be mindful of integrating user engagement research and co-design processes with potential users throughout the development phase to ensure that JITAIs are relevant and responsive to the population of interest. Furthermore, if users’ need for an adaptive feedback mechanism is adequately met, the burden of data entry will potentially be minimized over time as user will only need to input relevant information when required. Given the potential of wearables and smartphones to continuously track behaviors and acquire ecologically momentary information of individuals, DHIs should continue working towards interventions that are dynamic and adaptable to changing status and contexts^30^, and to the proffered communication channels (e.g., utilizing omnichannel communication)^31^.

Besides timely and adaptive feedback, women expect DHIs to be customized to their current pregnancy phase and circumstances (e.g., prior miscarriages, currently pregnant with twins, second-time mums). In the current study, participants identified the need to have better awareness and information of their own and child’s health to be better empowered in their health-related decision making. Part of an individual’s ability to make informed decisions about their health behaviors is adequate health literacy that is specific to their health context^32^. Adequate access and understanding of relevant health information is essential to reducing unhealthy health behaviours^33^. As recommended by the 2021 Lancet and Financial Times Commission, by 2030, all governments should implement civic and digital health literacy efforts, including co-creation of digital tools and health narratives to aid health education and combat disinformation^34^. In pregnant women, those with adequate health literacy levels tend to make more informed choices with regard to prenatal testing and food selection, and have better understanding of the dangers of smoking during pregnancy^33,35^. On the other hand, limited health literacy in pregnant women has been linked with negative beliefs regarding medicine, and beliefs that healthcare providers are responsible for the health of their child^33^. To achieve greater health equity in the age of digital health, innovators should consider determinants on the digital tool-level that may impact the broader use of the DHI developed. For instance, considerations may include implementing accessibility (e.g., support for multilanguage, support for low literacy through improving readability and complementary audiovisual features, accessibility of DHI through multiple platforms) and design (e.g., community-based participatory design, emphasizing tolerance for error and simplicity) strategies^36^. For health literacy to translate into empowerment, DHIs should focus on capacity building (i.e., individual’s capacity to use health information effectively) through factoring in individuals’ competences, subjective perceptions of health and health needs^37^.

Despite keen interest in a personalized DHI, there were mixed responses in relation to providing personal information. While some participants were willing to only provide basic demographic information, other participants expressed the necessity of incorporating medical records (e.g., blood glucose reading, ultrasound scans) into DHIs to allow for dynamic tailoring. This has been similarly observed in various groups of technology users (e.g., smart wearables, smart home speakers) where the personalization-privacy paradox remains a point of discussion^38,39^. Personalization has the capacity to increase or decrease user engagement with the technology depending on the perceived privacy risk versus benefits^40^. While different types of users (e.g., ambivalent, benefit-oriented) can express varying levels of perceived benefits and privacy concerns, trust remains one of the predictors for privacy attitudes^41^. For instance, users’ trust in a website significantly predicts their intention to share data with the website^41^. As current participants expressed trust in the government including health-related agencies, and healthcare professionals, DHI initiatives might benefit from collaborative partnerships with government agencies and hospitals, along with notable branding of these partnerships. It should however be noted that trust in government can be highly variable across countries and relating to this authority as a strategy for building trust of the DHI user may be limited to locations such as Singapore.

Participants from the current study are generally tech-savvy and were able to source information from online sources such as medical websites, forums and mobile phone apps. Nevertheless, the majority of participants struggled to find information that are tailored to the local context. Among the list of fertility or pregnancy or baby-related apps used by participants in this study, only two were developed locally (see Table 2). Singapore is a multicultural country with three major ethnic groups – Chinese, Malay and Indian, with each group maintaining their own traditional pregnancy or postbirth practices^23,42^. Specific to diet, different ethnic groups tend to have different diet practices during pregnancy and postbirth due to traditional cultural beliefs. For instance, Chinese women reported decreased seafood consumption postbirth due to its ‘cold’ and ‘poisonous’ properties, while Malay women reduced beef and eggs consumption due to beliefs that they inhibit general recovery following birth^43^. When faced with uncertainty about the effectiveness of traditional practices and the lack of avenues available to obtain verified information, women from the current study were inclined to remain cautious and follow traditional advices from family and friends despite negative or no effects. In a recent systematic review examining culture appropriateness of digital tools designed for pregnant women with gestational diabetes mellitus, it was reported that only two out of 12 DHIs offer culturally appropriate content^44^. Accordingly, providing women with access to culturally adapted practices and information presents as a key component when designing DHIs with an education component.

When faced with struggles and setbacks, women in the current study looked to find health-related support and reassurance from other women in similar situations online. This is in line with existing studies that reported a wide range of topics searched on online pregnancy forums, including physical symptoms, struggles with conception, pregnancy complications, baby’s health, nutrition in pregnancy and product recommendations^45,46^. In this study, women reported feeling a sense of connectedness and emotionally supported when reading other women’s stories and experiences, especially when the journey was atypical. Patient narrative is a compelling tool that can be effective in communicating health-related information, and influencing attitudes, judgement and behaviors (e.g., promote positive health behaviors, reduce prejudice, increase attitudes towards consumer products)^47^. Promisingly, interventions with patient narrative components have shown positive effect on patients’ well-being and mood^48–50^. Given the multi-modal nature of DHI, DHIs have the potential to explore digital storytelling of patient narratives through different forms (e.g., written, short-form videos, online chat). By leveraging digital storytelling as a strategy for knowledge translation and support, DHIs have the potential to enhance accessibility, education and community building for the targeted population^51^.

As the current study focused on women in Singapore, some of the findings may be specific to the Singapore context. As localization of DHIs to include the local culture and practices emerged as a significant theme, this might differ in other cultural/societal contexts. Nevertheless, the push towards co-design for DHI may be broadly impactful across a spectrum of contexts and may serve as an effective catalyst and advocate for realizing relevant solutions to the users. Despite significant differences observed between cultures in the intention to adopt technology (e.g., performance expectancy had greater influence on behavior intention of Swiss consumers than Chinese consumers, while social influence asserted more influence on behavior intention of Chinese consumers than Swiss consumers)^52^, a considerable amount of technology interactions are not tailored to specific economic or cultural groups of interest^53^. Accordingly, continued efforts should be made towards designing for inclusivity and equity through consideration of patient population, cultural and linguistic backgrounds, gender, and disabilities^53^.

Overall, the current study identified user needs and expectations of DHI for women throughout preconception, pregnancy and postpregnancy. Through this, two major concepts emerged – personalization and localization of DHIs. Despite the extensive collective experience of digital health tools in our study population, these two domains of development remain limited in currently used digital health tools within Singapore. When personalizing DHIs, DHI developers should consider including a two-way interactivity function by providing timely and adaptive feedback to women in response to their tracking data, and customizing information access according to phases and circumstances. Nevertheless, balance should be maintained between privacy risk and personal information collected to enable personalization. With growing concerns about the amount of personal data collected for the personalization of digital tools in particular, fertility and menstruation tracking applications, further work exploring the balance between data privacy and DHI personalization should be undertaken^54,55^. When localizing DHIs, access to local/context-relevant health-related information and resources, and connecting women to the community through narratives should be prioritized.

Pre-, during and postpregnancy presents as an opportune juncture to intervene and promote positive health-related behavior change^15^. Insights from the current study can serve as a foundation in the initial innovation process required to optimize DHI development and implementation for women in these phases. Inclusive design of digital health can positively influence health equity and the first step towards equity is to understand the populations that the health systems are serving^53^. Through conscious effort of engaging vulnerable and/or underserved communities, technology developers and designers can begin addressing cultural and gender gaps when building DHIs to ensure equitable accessibility and experience^53^. Moving forward, continued conversations with patients and other stakeholders (e.g., healthcare providers, families, government agencies) are needed to maintain the co-design process and ensure the development of context-relevant innovations that successfully address the needs and expectations of the intended population.

## Methods

The COnsolidated criteria for REporting Qualitative research (COREQ) checklist was utilized for reporting purposes^56^.

### Recruitment

We recruited women who are trying to conceive, currently pregnant and up to two years postbirth through purposive sampling from the National University Hospital (NUH) and the community in Singapore (Clinicaltrials.gov Identifier: NCT05099900). Potential participants responded to advertisements placed around the hospital and public places (e.g., bus stops, community centers). Based on the contact details provided in the advertisements, participants were able to call or email the research team if interested. The research team then provided an overview of the study and a copy of the participant information sheet via email to interested participants. Participants were screened based on the following inclusion criteria: i) English fluency; ii) aged 21 to 45 years; and iii) actively trying to conceive or currently in first to third trimester of pregnancy or have a child aged 0–2 years. Participants were not eligible for the study if they met any of the following exclusion criteria: i) evidence/diagnosis of cognitive impairment; ii) current diagnosis of psychiatric disorder; iii) significant hearing impairment; iv) women requiring or who had any form of assisted conception. Recruitment took place over a period of nine months (November 2021 to July 2022) and ended when data saturation was achieved for each group. No participants refused to participate or dropped out after consenting to the study.

### Data collection

This study adopted a qualitative approach to allow an in-depth exploration of women’s maternal and healthcare experiences throughout preconception to postbirth, and their digital health expectations. Prior to the interview, all participants completed an online questionnaire (Supplementary Methods) regarding their demographics. Following that, a 60 to 90-minute, semistructured interview was conducted either in-person or online, via Zoom video-conference, depending on participants’ preference. In-person interviews were conducted in participants’ homes, cafes, or the research lab’s meeting room. All interviews were conducted in English with at least two researchers present. The data collection team consisted of VVL, SV, WYN, NYL and QYL, all female and trained in qualitative interviewing and analysis. VVL and SV are postdoctoral fellows while WYN, NYL and QYL are research assistants with bachelor’s degrees. Relationship with participants were established through communication via email or phone call to complete screening questions and scheduling of the interview session. Participants were informed about the team’s reasons for doing the research and the researchers’ interests in the research topic. Informed consent was obtained prior to the session and all participants consented to audio-recording of the interview for transcription purposes. Open-ended questions were asked during the interview to explore participant experiences with their preconception, pregnancy or postbirth journey and digital health. Guiding topics for discussion during the interview are presented in Table 4, with this paper focusing on the digital health segment of the interview. No field notes were made during or after the interview.

**Table 4:** Interview topic guide.

Topic	Guide for discussion
Women’s perspectives	Experiences in their current phase (challenges, positives)
	Phase-related changes (lifestyle, information-seeking, support system)
	Touchpoints with the healthcare system (for women and/or their children)
	Current quality of care and expectations of the healthcare system
Technology usage	Experience with digital health (e.g., wearables, mobile phone apps)
	Opinions on current digital health tools for preconception, pregnancy or postbirth
	Ideal features for digital health tools for preconception, pregnancy or postbirth
	Motivators and barriers to adoption and sustained usage for preconception, pregnancy or postbirth digital health tools

Following the interview, participants completed a questionnaire (Supplementary Methods) regarding their pregnancy concerns and digital health expectations. All participants were also reimbursed for their time (Singapore Dollar (SGD)15/~United States Dollar (USD)11.27 for 1 h of interview and SGD20/ ~ USD15.03 for transport). No repeat interviews were conducted and transcripts were not returned to the participants. The study procedures were approved by the National Healthcare Group Domain Specific Review Board (DSRB; DSRB reference number: 2021/00034). All data collected, including signed consent forms, interview recordings and questionnaire data, were deidentified, encrypted and stored in a secure database.

### Data analysis

All of the interview recordings were transcribed verbatim and inductive thematic analysis was used to identify emerging themes. Firstly, primary coding, during which data were descriptively labelled, was conducted collectively by five researchers (VVL, SV, WYN, QYL, NYL). Transcripts were randomly assigned to one of the five researchers and independently coded. Primary codes generated from all researchers were compared and discussed to resolve any discrepancies. Subsequently, secondary coding, where labelled data were grouped into categories, was conducted in Microsoft Excel. The initial framework of categories was developed by VVL and SV, and discussed with the study analysis team for further input. Categories that emerged from secondary coding were analyzed and grouped into broader, overarching themes^57^. The final set of codes and broader themes were generated from discussions and iterations by the study analysis team. Participants did not provide feedback on the findings.

Thematic analysis was conducted and reported with study data as a whole. In the early stages of data analysis, categorizations were conducted in independent groupings however, similar themes emerged across all three groups (i.e., preconception, pregnancy and postbirth). Despite minor variations in information women seek (e.g., fertility-related versus pregnancy-related), it is important to note that all themes and subthemes recurred in all three groups of women.

### Reporting summary

Further information on research design is available in the Nature Research Reporting Summary linked to this article.

### Supplementary information

Supplementary methods

Reporting Summary

## Data Availability

The datasets used and/or analyzed during the current study available from the corresponding author on reasonable request.
