# Supplementary material for: Personalization and localization as key expectations of digital health intervention in women pre- to post-pregnancy
Source: NPJ Digit Med. 2023 Sep 30;6:183. doi: 10.1038/s41746-023-00924-6 (PMC10541409; doi:10.1038/s41746-023-00924-6)
Supplement: Supplementary file 1 — Supplementary methods [file 41746_2023_924_MOESM1_ESM.pdf]

Questions from the pre-and post-interview questionnaires

1. What is your current status of employment?
    - Employed full-time
    - Employed part-time
    - Unemployed
    - Others – please state: _____

2. What is your current occupation?

3. What type of housing are you currently living in?
    - 1- tor 2-room public housing
    - 3-, 4- or 5-room public housing
    - Private housing (e.g., condominiums, landed properties, etc.)

4. On average, how much time do you spend away from work in a day (e.g., on hobbies, relaxation, etc.)?
    - Less than 2 hours
    - Between 2 to 5 hours
    - More than 5 hours
    - N/A

5. How often do you engage in physical activities (e.g., exercise) in a week?
    - 0 to 2 times a week
    - 3 or 4 times a week

    a) 5 or more times a week

6. Please rank your sources of support during this time, e.g., pregnancy, childcare (1 being the highest).
    - Partner
    - Parent(s)
    - Sibling(s)
    - Extended family
    - Friends
    - Community
    - Healthcare system

7. Please rank the following types of assistance you find useful to have during this time (1 being the highest).
    - Advice on mental health
    - Advice on physical health
    - Advice on lifestyle (e.g., diet)
    - Parenting tips
    - Medical-related advice for mothers/mothers-to-be
    - Medical-related advice for child

8. Do you use the Internet to search for pregnancy/maternal-related information or advice?
    - Yes
    - No

9. Which of the following online sources do you find useful?
   - Chat groups (e.g., Telegram, WhatsApp chat groups)
   - Online forums
   - Google search (e.g., Wikipedia)
   - Mobile phone apps
   - Social media platforms (e.g., Instagram, Facebook, YouTube)
   - None, I don't find online resources useful.
   - Others – please state: ___________

10. Please rank the following in the order you would seek for pregnancy/maternal-related information or advice (1 being the highest).
    - Internet (e.g., Google, websites, media articles, online forums)
    - Mobile phone apps
    - Social media platforms (e.g. Instagram, Facebook, YouTube)
    - Healthcare professionals (e.g., doctors, nurses, etc.)
    - Alternative health practitioners (e.g., TCM, Ayurveda)
    - Family
    - Friends

11. What are the health apps (e.g., fitness, lifestyle, pregnancy, childcare-related apps) you use generally? Please list: ___

12. What are some of the pregnancy-related concerns that have crossed your mind before? (Select all applicable)
    - Fear of gaining weight
    - Pre-/post-partum depression
    - Anxiety from trying to get pregnant or the thought of childcare
    - Stretch marks
    - Others – please state: ___________________

13. How likely would you use a digital health platform that:

i) requires completing standard questionnaires (e.g., food intake, physical activity and psychological health) at regular intervals
   - Extremely likely
   - Somewhat likely
   - Neutral
   - Somewhat unlikely
   - Extremely unlikely

ii) offers the function of logging in physical health data (e.g., weight, diet and physical activity) for the purpose of tracking and monitoring
   - Extremely likely
   - Somewhat likely
   - Neutral
   - Somewhat unlikely
   - Extremely unlikely

iii) offers the function of logging in mental health data (e.g., stress, mood) for the purpose of tracking and monitoring

- Extremely likely
- Somewhat likely
- Neutral
- Somewhat unlikely
- Extremely unlikely

iv) sends feedback to you whenever you key in your physical and/or mental health information

- Extremely likely
- Somewhat likely
- Neutral
- Somewhat unlikely
- Extremely unlikely

v) offers lifestyle guidelines and advices

- Extremely likely
- Somewhat likely
- Neutral
- Somewhat unlikely
- Extremely unlikely

vi) offers peer support

- Extremely likely
- Somewhat likely
- Neutral
- Somewhat unlikely
- Extremely unlikely

vii) offers information on breastfeeding and weaning

- Extremely likely
- Somewhat likely
- Neutral
- Somewhat unlikely
- Extremely unlikely

viii) can be connected to wearable devices to track activities (e.g., weight, steps taken, sleep)

- Extremely likely
- Somewhat likely
- Neutral
- Somewhat unlikely
- Extremely unlikely

ix) can be paired with digital tools (e.g., Bluetooth-enabled weighing machine) for the ease of tracking and monitoring

- Extremely likely
- Somewhat likely
- Neutral
- Somewhat unlikely

- Extremely unlikely

14. Which of the following frequency is acceptable to you with regards to logging in of information?
    - Daily
    - Weekly
    - Monthly
    - Others – please state: ____________________

15. What type of topics do you wish to be able to access in a digital health platform?
    - Developmental information of the foetus/baby
    - Mental health resources
    - Physical activity ideas and videos
    - Helpline and health provider contact details
    - Others – please state: ____________________

16. I prefer to stay anonymous when interacting with peers on a digital health platform
    - Strongly agree
    - Agree
    - Neutral
    - Disagree
    - Strongly disagree
