# Supplementary material for: Personalization and localization as key expectations of digital health intervention in women pre- to post-pregnancy
Source: NPJ Digit Med. 2023 Sep 30;6:183. doi: 10.1038/s41746-023-00924-6 (PMC10541409; doi:10.1038/s41746-023-00924-6)
Supplement: Supplementary file 2 — Reporting Summary [file 41746_2023_924_MOESM2_ESM.pdf]

nature portfolio

Corresponding author(s): V Vien Lee, Yung Seng Lee, Shiao-Yng Chan, Agata Blasiak, Dean Ho

Last updated by author(s): Aug 15, 2023

Reporting Summary

Nature Portfolio wishes to improve the reproducibility of the work that we publish. This form provides structure for consistency and transparency in reporting. For further information on Nature Portfolio policies, see our Editorial Policies and the Editorial Policy Checklist.

Statistics

For all statistical analyses, confirm that the following items are present in the figure legend, table legend, main text, or Methods section.

n/a	Confirmed	
☐	☒	The exact sample size (*n*) for each experimental group/condition, given as a discrete number and unit of measurement
☐	☒	A statement on whether measurements were taken from distinct samples or whether the same sample was measured repeatedly
☒	☐	The statistical test(s) used AND whether they are one- or two-sided<br>*Only common tests should be described solely by name; describe more complex techniques in the Methods section.*
☒	☐	A description of all covariates tested
☒	☐	A description of any assumptions or corrections, such as tests of normality and adjustment for multiple comparisons
☒	☐	A full description of the statistical parameters including central tendency (e.g. means) or other basic estimates (e.g. regression coefficient) AND variation (e.g. standard deviation) or associated estimates of uncertainty (e.g. confidence intervals)
☒	☐	For null hypothesis testing, the test statistic (e.g. *F*, *t*, *r*) with confidence intervals, effect sizes, degrees of freedom and *P* value noted<br>*Give P values as exact values whenever suitable.*
☒	☐	For Bayesian analysis, information on the choice of priors and Markov chain Monte Carlo settings
☒	☐	For hierarchical and complex designs, identification of the appropriate level for tests and full reporting of outcomes
☒	☐	Estimates of effect sizes (e.g. Cohen's *d*, Pearson's *r*), indicating how they were calculated

Our web collection on statistics for biologists contains articles on many of the points above.

Software and code

Policy information about availability of computer code

Data collection	Microsoft Excel and Word were used during the data collection process
Data analysis	Microsoft Excel and Word were used during the data analysis process

For manuscripts utilizing custom algorithms or software that are central to the research but not yet described in published literature, software must be made available to editors and reviewers. We strongly encourage code deposition in a community repository (e.g. GitHub). See the Nature Portfolio guidelines for submitting code & software for further information.

Data

Policy information about availability of data

All manuscripts must include a data availability statement. This statement should provide the following information, where applicable:

- Accession codes, unique identifiers, or web links for publicly available datasets
- A description of any restrictions on data availability
- For clinical datasets or third party data, please ensure that the statement adheres to our policy

The datasets used and/or analyzed during the current study available from the corresponding author on reasonable request.

Research involving human participants, their data, or biological material

Policy information about studies with human participants or human data. See also policy information about sex, gender (identity/presentation), and sexual orientation and race, ethnicity and racism.

Reporting on sex and gender	The study explored the needs and expectations of women in three different phases: pre-conception, pregnancy and post-birth. All participants identified as women. Sex was determined based on self-reporting, i.e. responding to the study advert specifying the recruitment of women in the pre-pregnancy phase (trying to conceive), currently pregnant or in the post-pregnant phase (with a child aged 0-2 years).
Reporting on race, ethnicity, or other socially relevant groupings	Ethnicity and socioeconomic status data were collected and reported as we wanted to ensure that the current study captured the experiences of a diverse group of participants that closely reflect the Singapore population. In the screening questionnaire, participants were asked about their ethnicity and monthly household income. Participants selected the ethnicity (i.e., Chinese, Malay, Indian, Others (please list)) they identify with on the questionnaire. Low (< SGD 3,999), middle (SGD 4,000-9,999) and high (> SGD 10,000) socioeconomic status were determined based on the monthly household income declared by the participants. The reported median monthly household income in 2020 was SGD 7,744.
Population characteristics	See below.
Recruitment	The current study recruited women who are trying to conceive, currently pregnant and up to two years post-birth through purposive sampling from the National University Hospital (NUH) and the community in Singapore. Potential participants responded to advertisements placed around the hospital and public places (e.g., bus stops, community centers). Based on the contact details provided in the advertisements, participants were able to call or email the research team if interested.
Ethics oversight	National Healthcare Group Domain Specific Review Board (DSRB; DSRB reference number: 2021/00034) approved the study protocol.

Note that full information on the approval of the study protocol must also be provided in the manuscript.

Field-specific reporting

Please select the one below that is the best fit for your research. If you are not sure, read the appropriate sections before making your selection.

☐ Life sciences ☒ Behavioural & social sciences ☐ Ecological, evolutionary & environmental sciences

For a reference copy of the document with all sections, see nature.com/documents/nr-reporting-summary-flat.pdf

Behavioural & social sciences study design

All studies must disclose on these points even when the disclosure is negative.

Study description	The current study is a qualitative study, which utilized semi-structured interviews to explore women's maternal, healthcare and technology experiences throughout pre-conception, pregnancy and post-birth.
Research sample	A total of 44 women (age range = 21-40; mean age = 31.6 years, SD = 4.0) completed the study. Similar to Singapore's ethnic distribution, participants were of Chinese (75.0%), Malay (9.1%), Indian (9.1%) and other (6.8%) ethnicities, and all participants spoke fluent English. The study population included women from three different groups: i) trying to conceive, ii) currently pregnant, and iii) up to two years post-birth. These groups of women were selected as the study was interested in women's digital health intervention needs and expectations before, during and after pregnancy.
Sampling strategy	We recruited women who are trying to conceive, currently pregnant and up to two years post-birth through purposive sampling from the National University Hospital (NUH) and the community in Singapore. Potential participants responded to advertisements placed around the hospital and public places (e.g., bus stops, community centers). Data saturation was considered - data collection ended when new interviews produced little or no change to the themes.
Data collection	Prior to the interview, all participants completed an online questionnaire regarding their demographics. Following that, a 60 to 90-minute, semi-structured interview was conducted either in-person or online (via Zoom), depending on participants' preference. In-person interviews were conducted in participants' homes, cafes, or the research lab's meeting room. All interviews were conducted in English with at least two researchers present. Informed consent was obtained prior to the session and all participants consented to audio-recording of the interview for transcription purposes. Following the interview, participants completed an online questionnaire regarding their pregnancy concerns and digital health expectations. All participants were reimbursed for their time.
Timing	Recruitment and data analysis took place from November 2021 to July 2022.
Data exclusions	No data were excluded from the study.
Non-participation	One hundred and twenty-five women indicated interest in the study but 81 were not enrolled in the study due to purposive sampling strategies (30 individuals), lack of response from participants after receiving the information sheet (22 individuals), and the study's pre-determined inclusion and exclusion criteria (3 individuals).

Randomization	There was no experimental group in the study. Accordingly, no participants were allocated in experimental groups.

Reporting for specific materials, systems and methods

We require information from authors about some types of materials, experimental systems and methods used in many studies. Here, indicate whether each material, system or method listed is relevant to your study. If you are not sure if a list item applies to your research, read the appropriate section before selecting a response.

Materials & experimental systems

n/a	Involved in the study
☒	☐ Antibodies
☒	☐ Eukaryotic cell lines
☒	☐ Palaeontology and archaeology
☒	☐ Animals and other organisms
☒	☐ Clinical data
☒	☐ Dual use research of concern
☒	☐ Plants

Methods

n/a	Involved in the study
☒	☐ ChIP-seq
☒	☐ Flow cytometry
☒	☐ MRI-based neuroimaging
